# Exploratory Label-Free Proteomic Profiling of the *Escherichia coli* Response to Sub-Inhibitory CORM-3 Exposure

**DOI:** 10.3390/microorganisms14091918

**Published:** 2026-08-31

**Authors:** Salar Ali, Salvatore Dimonte, Stefano Aquaro, Muhammed Babakir-Mina, Giovanni Di Bonaventura, Arianna Pompilio

**Affiliations:** 1 College of Health and Medical Technology, Sulaimani Polytechnic University, Sulaimani 46001, Iraq; 2 Nursing Department, Kurdistan Technical Institute, Sulaimani 46001, Iraq; 3 Department of Molecular Biology and Biotechnology, The University of Sheffield, Sheffield S10 2TN, UK; 4 BioMolecular Lab, 76121 Barletta, Italy; 5 Department of Life, Health and Environmental Sciences, University of L’Aquila, 67100 L’Aquila, Italy; 6 Department of Medical, Oral, and Biotechnological Sciences, “G. d’Annunzio” University of Chieti-Pescara, 66100 Chieti, Italy; gdibonaventura@unich.it (G.D.B.); arianna.pompilio@unich.it (A.P.); 7 Center for Advanced Studies and Technology (CAST), “G. d’Annunzio” University of Chieti-Pescara, 66100 Chieti, Italy

**Keywords:** carbon monoxide-releasing molecules, CORM-3, *Escherichia coli*, label-free proteomics, bacterial stress response

## Abstract

Carbon monoxide-releasing molecules (CORMs) display promising antibacterial activity, yet their global cellular targets and underlying mechanisms remain incompletely understood. This study aimed to characterize the proteomic response of *Escherichia coli* MG1655 exposed to a sub-inhibitory concentration (10 µM, 40 min) of the ruthenium-based CORM-3 compared to its inactive counterpart, iCORM-3, in defined minimal medium. Utilizing label-free quantitative LC-MS/MS proteomics combined with differential abundance, functional annotation, and STRING-based network analyses, we explored treatment-associated proteomic signatures and subcellular localization patterns. While iCORM-3 exhibited no antibacterial activity, CORM-3 inhibited growth in a concentration-dependent manner. Although no individual protein reached FDR significance after multiple-testing correction, candidate abundance changes converged on functionally related modules, including envelope/periplasmic stress, sulfur metabolism and transport, redox/metal homeostasis and selected energy-associated functions. These findings support a hypothesis-generating model in which sub-inhibitory CORM-3 exposure is associated with multifactorial bacterial stress adaptation rather than a single dominant protein-level target. Envelope homeostasis, sulfur-containing pathways, redox/metal adaptation, and attenuation of selected energy-associated functions therefore emerge as candidate components of the *E. coli* response to CORM-3 and require future targeted validation.

## 1. Introduction

Carbon monoxide (CO) has long been recognized as an inhibitor of aerobic respiration, primarily by binding heme-containing proteins, including hemoglobin and cytochrome c oxidase, thereby interfering with oxygen transport and mitochondrial electron transport chain activity [1]. At low concentrations, however, endogenous CO produced by heme oxygenases has been implicated in several physiological and cytoprotective processes, including anti-inflammatory, vasodilatory and anti-apoptotic effects, and is now considered part of a broader gasotransmitter biology framework [2]. Nevertheless, the administration of CO gas as a therapeutic agent remains challenging because of its toxicity and the difficulty of achieving controlled systemic delivery [3].

Therefore, CO-releasing molecules (CORMs) have been developed over the past two decades as pharmacological tools to deliver controlled amounts of CO while reducing the limitations associated with CO gas administration [4]. Several chemically distinct classes of CO-releasing molecules have been described, including transition metal carbonyl complexes based on ruthenium, iron and manganese, as well as non-metallic CO donors such as boranocarbonate-based compounds [4]. Their CO-release rates and kinetics vary with the donor molecule’s chemical structure and activation mechanism, with reported half-lives ranging from seconds to hours [4]. Accordingly, CORMs can release CO through spontaneous or chemically triggered dissociation in biological media, as well as through enzyme-triggered activation in specifically designed CORM prodrugs [5,6].

CORM-3 is a ruthenium-based, water-soluble compound [Ru(CO)_3_Cl (glycinate)] that releases one CO molecule per compound molecule with a half-life of around 2 min in vitro [7,8]. CORM-3 has shown bactericidal properties toward Gram-negative and Gram-positive bacteria, including *Escherichia coli*, *Pseudomonas aeruginosa*, and *Staphylococcus aureus* [5,9,10,11,12].

To discriminate biological effects attributable to CO release from those potentially associated with the CO-depleted Ru-containing compound, it is essential to use the inactivated counterpart, iCORM-3, as a control compound. However, a number of studies did not use iCORMs as a control [5,9,10,11,12] or utilized controls that are not the inactivated compound, but a similar compound where CO has been substituted for another molecule, such as RuCl_2_(DMSO)_4_ [9].

In this work, iCORM-3 was prepared by incubating fresh CORM-3 dissolved in phosphate-buffered saline (PBS) at room temperature for up to 48 h with periodic nitrogen bubbling, after which very little CO was released (less than 5% of the amount released at the same CORM-3 concentration). This process is thought to allow CORM-3 to release the labile CO, which escapes into the gas phase upon bubbling, leaving a CO-depleted CORM-3 compound in solution.

The interpretation of CORM-3 activity is further complicated by evidence that its biological effects may not depend exclusively on CO release. Indeed, Southam et al. showed that a thiol-reactive Ru (II) species can make a major contribution to the antimicrobial and cytotoxic properties of CORM-3 [13]. Therefore, the use of iCORM-3 as a CO-depleted comparator remains important to distinguish responses associated with the active CO-releasing molecule from those potentially related to the ruthenium-containing scaffold.

Despite the ambiguity of its chemical structure, iCORM-3 has been used as a non-CO-releasing control for CORM-3 to assess the contribution of the Ru-containing compound after removal of labile CO. Previous transcriptomic work showed that iCORM-3 was less effective than CORM-3 and provided a comprehensive analysis of bacterial responses to both compounds [14].

Building on these findings, the present study employs a proteomic approach to examine *E. coli* exposed to CORM-3 or iCORM-3, to define protein-level responses associated with each stress condition, and to compare them with previously reported transcriptomic signatures. This approach may help identify cellular processes targeted by CORM-3 and provide further insights into its antibacterial mode of action.

## 2. Materials and Methods

CORM-3 (tricarbonylchloro (glycinate) ruthenium (II)) was obtained from Prof. Brian Mann (Department of Chemistry, University of Sheffield, UK). Stock solutions (10 mM) were prepared in water and stored on ice. The stock solution was used fresh or the following day after overnight storage at 4 °C.

For iCORM-3 preparation, CORM-3 was dissolved in PBS to a final concentration of 10 mM. The solution was sparged with nitrogen for 5 min immediately after preparation and then for 5 min every 2 h the following day [7,8]. On the day of use, the solution was sparged with nitrogen once more for 5 min.

### 2.1. Bacterial Strain and Growth Conditions

Starter cultures of *E. coli* MG1655 were grown in 10 mL of Luria–Bertani (LB) broth overnight, and then LB was removed by centrifugation (5500× *g* for 5 min at 4 °C). The cells were then resuspended in defined minimal medium (DMM) containing 54 mM glycerol, as the sole and limiting energy and carbon source, 23 mM K_2_HPO_4_, 7.3 mM KH_2_PO_4_, 18.7 mM NH_4_Cl, 69 μM CaCl_2_, 15 mM K_2_SO_4_, 1 mM MgCl_2_, and the following trace elements in 134 μM EDTA: 31 μM FeCl_3_·6H_2_O, 6.15 μM ZnO, 570 nM CuCl_2_·2H_2_O, 340 nM CoNO_3_·6H_2_O, 1.6 μM H_3_BO_3_ (all final concentrations) in distilled H_2_O [15]. After sterilization by autoclaving, the pH of the medium was maintained at 7.0 by adding sterile 1 M NaOH or HCl. The cultures were kept at 37 °C with shaking at 200 rpm. CORM-3 or inactivated CORM-3 (iCORM-3) was added to the cultures when the OD_600_ reached ≈ 0.45.

### 2.2. Antibacterial Activity Assay

Bacterial cells were grown in DMM, as previously described, to the exponential phase (OD_600_: 0.4–0.5). Cells were then challenged with CORM-3 (10 μM, 20 μM, 30 μM, and 40 μM) and iCORM-3 (40 μM). Cultures were incubated at 37 °C with shaking at 200 rpm, and OD_600_ values were measured every 2 h for 24 h.

### 2.3. Experimental Design for Proteomic Analysis

**Figure 1:**
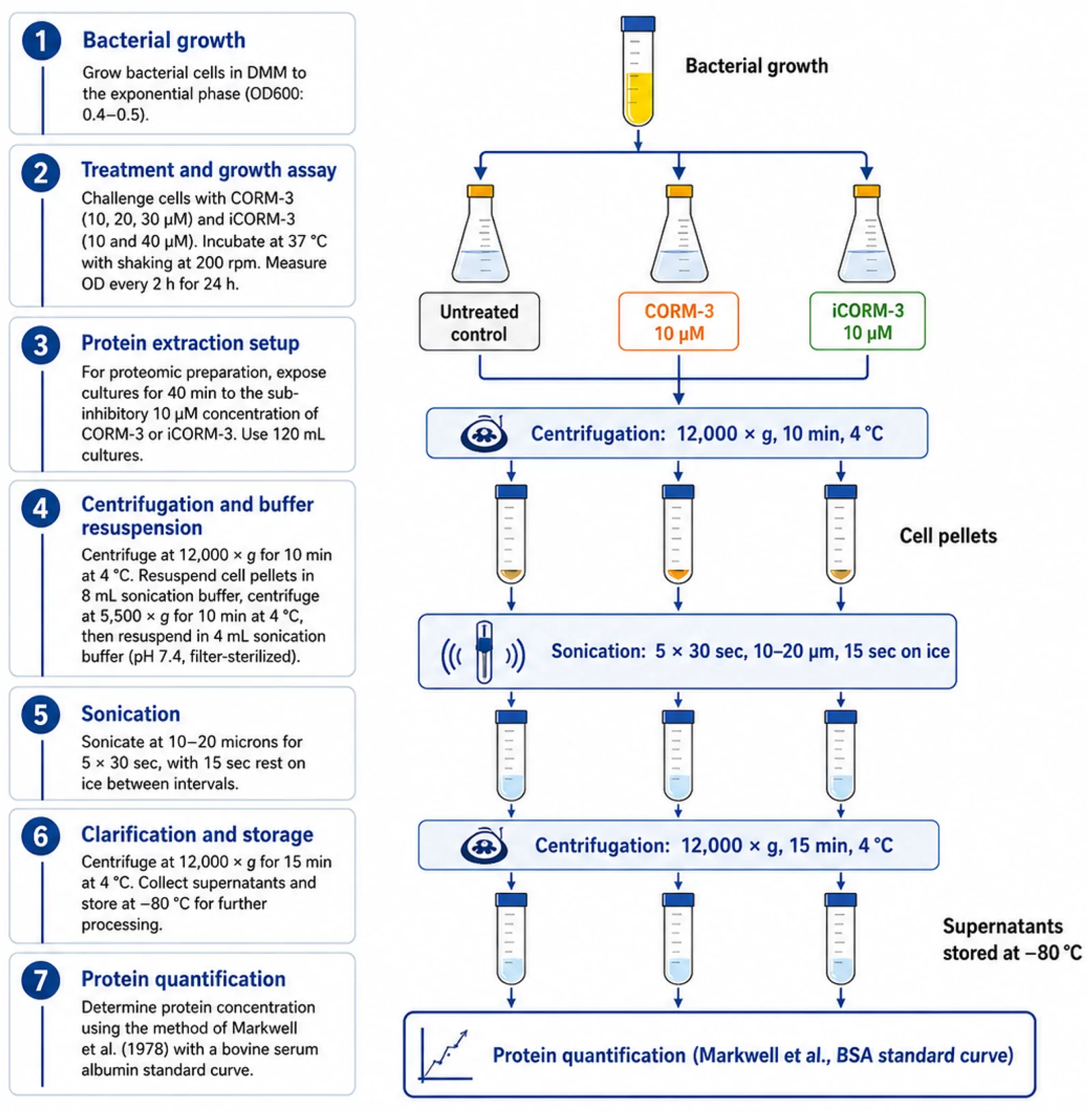
Experimental workflow for CORM-3-based proteomic analysis. *E. coli* MG1655 cultures were exposed for 40 min to 10 µM CORM-3 or to 10 µM iCORM-3 as the inactive comparator, with untreated cultures processed as baseline controls. Cells were then collected for protein extraction, quantification, and downstream LC-MS/MS analysis [16].

For proteomic analysis, bacterial cultures were exposed to 10 µM CORM-3 for 40 min, corresponding to the selected sub-inhibitory concentration. Cultures exposed to 10 µM iCORM-3 were processed in parallel as inactive-compound controls, and untreated cultures were included as baseline controls. For each condition, 120 mL of bacterial culture was collected and processed as described below. The experimental workflow is summarized in Figure 1.

### 2.4. Protein Extraction and Quantification

One hundred and twenty milliliters of untreated or treated bacterial culture were centrifuged at 12,000× *g* for 10 min at 4 °C. Cell pellets were washed by resuspension in 8 mL of sonication buffer (Tris-HCl 7.88 g, MgCl_2_ 395.8 mg and EGTA 380.35 mg in 1 L of distilled water; pH adjusted to 7.4 and filter-sterilized), centrifuged at 5500× *g* for 10 min at 4 °C, and finally resuspended in 4 mL of sonication buffer containing protease inhibitor cocktail (Sigma-Aldrich, St. Louis, MO, USA). The suspensions were sonicated at 10–20 μm amplitude for five 30-s intervals, with 15-s rests on ice between intervals. After centrifugation at 12,000× *g* for 15 min at 4 °C, supernatants were stored at −80 °C until further processing. Protein concentration was determined according to Markwell et al. [16], using bovine serum albumin as a standard. Protein extracts were then normalized to the same concentration before electrophoretic separation.

### 2.5. One-Dimensional SDS-PAGE

One hundred micrograms of protein extracts were mixed with sample buffer containing TCEP at a final concentration of 1 mM and incubated at 70 °C for 10 min. After cooling to room temperature, samples were alkylated with iodoacetamide at a final concentration of 2 mM for 30 min in the dark. Samples were then loaded onto an SDS-PAGE gel and subjected to electrophoresis at 150 V for 45 min. The gel was fixed with 40% methanol/2% acetic acid for 30 min, stained overnight with colloidal Coomassie, and destained with 25% methanol for 1–2 h (Amersham Biosciences, Waukesha, WI, USA).

### 2.6. In-Gel Digestion and Peptide Extraction

Coomassie-stained gel pieces were destained with 500 μL of 50% acetonitrile in 50 mM ammonium bicarbonate, pH 8.5, under gentle shaking at 600 rpm for 2 h at room temperature. Gel pieces were then dehydrated with 100 μL of 100% acetonitrile for 15 min at room temperature, the solvent was removed, and the samples were briefly dried temperature (Promega Corporation, Madison, WI, USA). Sequencing-grade trypsin was prepared at 20 µg/mL in 50 mM ammonium bicarbonate, and 150 μL of trypsin solution was added to each tube containing dried gel pieces. Proteolytic digestion was performed at 37 °C for 1 h in a thermomixer under gentle shaking at 600 rpm, followed by overnight incubation at 25 °C. Peptides were extracted by sequential incubations with acetonitrile and 0.5% formic acid. Briefly, 100 μL of acetonitrile was added to each tube, which was incubated at 37 °C for 15 min under gentle shaking, and the supernatant was transferred to a peptide collection tube. Gel pieces were then incubated with 50 μL of 0.5% formic acid at 37 °C for 15 min, followed by the addition of 100 μL of acetonitrile and a further 15-min incubation at 37 °C. This formic acid/acetonitrile extraction was repeated once, followed by a final extraction with 100 μL of acetonitrile (Sigma-Aldrich, St. Louis, MO, USA). Peptide-containing supernatants from the same sample were pooled, dried in a vacuum concentrator, and stored at −20 °C until LC-MS/MS analysis.

### 2.7. LC-MS/MS Analysis, Database Search, and Protein Identification

Extracted peptides were resuspended in 0.5% formic acid and analyzed by nano-liquid chromatography–tandem mass spectrometry (nanoLC-MS/MS) using an Orbitrap Elite hybrid mass spectrometer (Thermo Fisher Scientific, Waltham, MA, USA) equipped with a nanospray source and coupled to an Ultimate RSLCnano LC system (Dionex, Sunnyvale, CA, USA). The system was controlled using Xcalibur 2.1 (Thermo Fisher Scientific, Waltham, MA, USA) and DCMSLink 2.08 (Dionex, Sunnyvale, CA, USA). Peptide samples were desalted online using a C18 PepMap 100 micro-precolumn cartridge and separated on an Easy-Spray PepMap C18 analytical column (15 cm × 50 μm inner diameter, 2 μm particle size, 100 Å pore size; Thermo Fisher Scientific, Waltham, MA, USA). Peptides were eluted using a 60-min reversed-phase gradient from 4% to 32% solvent B, where solvent A was 0.1% formic acid in water and solvent B was acetonitrile containing 0.1% formic acid. The Orbitrap Elite was operated in data-dependent acquisition mode. Full MS scans were acquired in the Orbitrap at a resolution of 60,000 at *m*/*z* 400, and the 20 most abundant multiply charged precursor ions with charge state ≥ 2 were selected for MS/MS fragmentation in the linear ion trap. The FTMS target value was 1 × 10^6^, and the ion trap MS/MS target value was 1 × 10^4^, with lock mass enabled at *m*/*z* 445.120025. Maximum accumulation times were set to 500 ms for FTMS scans and 100 ms for ion trap MS/MS scans. Dynamic exclusion was enabled with a repeat duration of 45 s, an exclusion list size of 500 and an exclusion duration of 30 s. Raw MS files were processed using MaxQuant version 1.5.2.8 and searched against the UniProt *E. coli* K-12 MG1655 protein database. Trypsin was selected as the digestion enzyme, allowing up to two missed cleavages. Precursor and fragment ion mass tolerances were set to 7 ppm and 0.5 Da, respectively. Carbamidomethylation of cysteine was set as a fixed modification, whereas methionine oxidation and protein N-terminal acetylation were set as variable modifications. Peptide and protein identifications were accepted at a 1% false discovery rate (FDR) at both levels.

### 2.8. Label-Free Quantification and Comparative Proteomic Analysis

Label-free quantification was performed using MaxQuant-calculated LFQ protein intensities. The match-between-runs option was enabled with a 2-min retention-time window to improve peptide identification transfer between LC-MS/MS runs. Reverse hits, potential contaminants and proteins identified only by site were removed before downstream analysis. The resulting LFQ intensity matrix included untreated controls, CORM-3-treated cells and iCORM-3-treated cells, with three independent biological replicates per condition. LFQ intensities were log2-transformed and used for quality control, differential abundance analysis, heatmap visualization and functional interpretation. Pairwise comparisons were performed for CORM-3 versus untreated control, iCORM-3 versus untreated control and CORM-3 versus iCORM-3, as detailed in Section 2.10.

### 2.9. Functional Annotation and Enrichment Analysis

Candidate DAPs were functionally interpreted using Gene Ontology (GO), KEGG pathway, COG functional category and STRING protein–protein interaction analyses. Separate protein lists were generated for each pairwise comparison: CORM-3 versus untreated control, iCORM-3 versus untreated control and CORM-3 versus iCORM-3. Proteins with increased or decreased abundance were analyzed separately when appropriate. GO enrichment analysis considered Biological Process, Molecular Function and Cellular Component categories. KEGG pathway analysis was used to identify metabolic and regulatory pathways associated with treatment-responsive proteins, whereas COG classification summarized candidate proteins across broad functional categories. Protein–protein interaction networks were generated using STRING with *E. coli* K-12 MG1655 as the reference organism. Network nodes represented proteins, whereas edges represented functional associations. Enrichment results were corrected for multiple testing, and terms with adjusted *p*-value or FDR < 0.05 were considered enriched. Given the exploratory nature of the differential abundance analysis, functional results were interpreted as hypothesis-generating and used to identify biological processes most consistently associated with CORM-3 exposure.

### 2.10. Statistical Analysis

Growth-curve data are reported as mean ± standard deviation from three independent biological replicates, and doubling time was estimated by nonlinear regression in GraphPad Prism version 9.4.0 (GraphPad Software, USA). Proteomic analyses used label-free quantification (LFQ) intensity values from three independent biological replicates for each condition: untreated control, CORM-3-treated cells, and iCORM-3-treated cells. Zero intensity values were treated as missing values. For quality control, LFQ intensities were log2-transformed and used to assess protein recovery per sample, intensity distributions, sample-to-sample Pearson correlations, and principal component analysis (PCA). PCA was performed on proteins quantified in at least five of nine samples after median imputation of missing values and variable standardization. Differential abundance analysis was performed on log2-transformed LFQ intensities for three pairwise comparisons: CORM-3 versus untreated control, iCORM-3 versus untreated control, and CORM-3 versus iCORM-3. For each protein, log2 fold changes were calculated from mean LFQ intensities between groups, and statistical significance was assessed using Welch’s *t*-test. *p*-values were adjusted for multiple testing using the Benjamini–Hochberg procedure to estimate the FDR. Proteins were considered statistically significant when reaching FDR < 0.05. For exploratory biological interpretation, candidate differentially abundant proteins were defined as those with a nominal *p*-value < 0.05 and |log2FC| ≥ 1, corresponding to a fold change ≥ 2 or ≤0.5. Heatmaps were generated from row-normalized z-scores calculated from log2 LFQ intensities, with median imputation used only for visualization.

## 3. Results

### 3.1. Determination of a Sub-Inhibitory Concentration of CORM-3 on E. coli Growth

**Figure 2:**
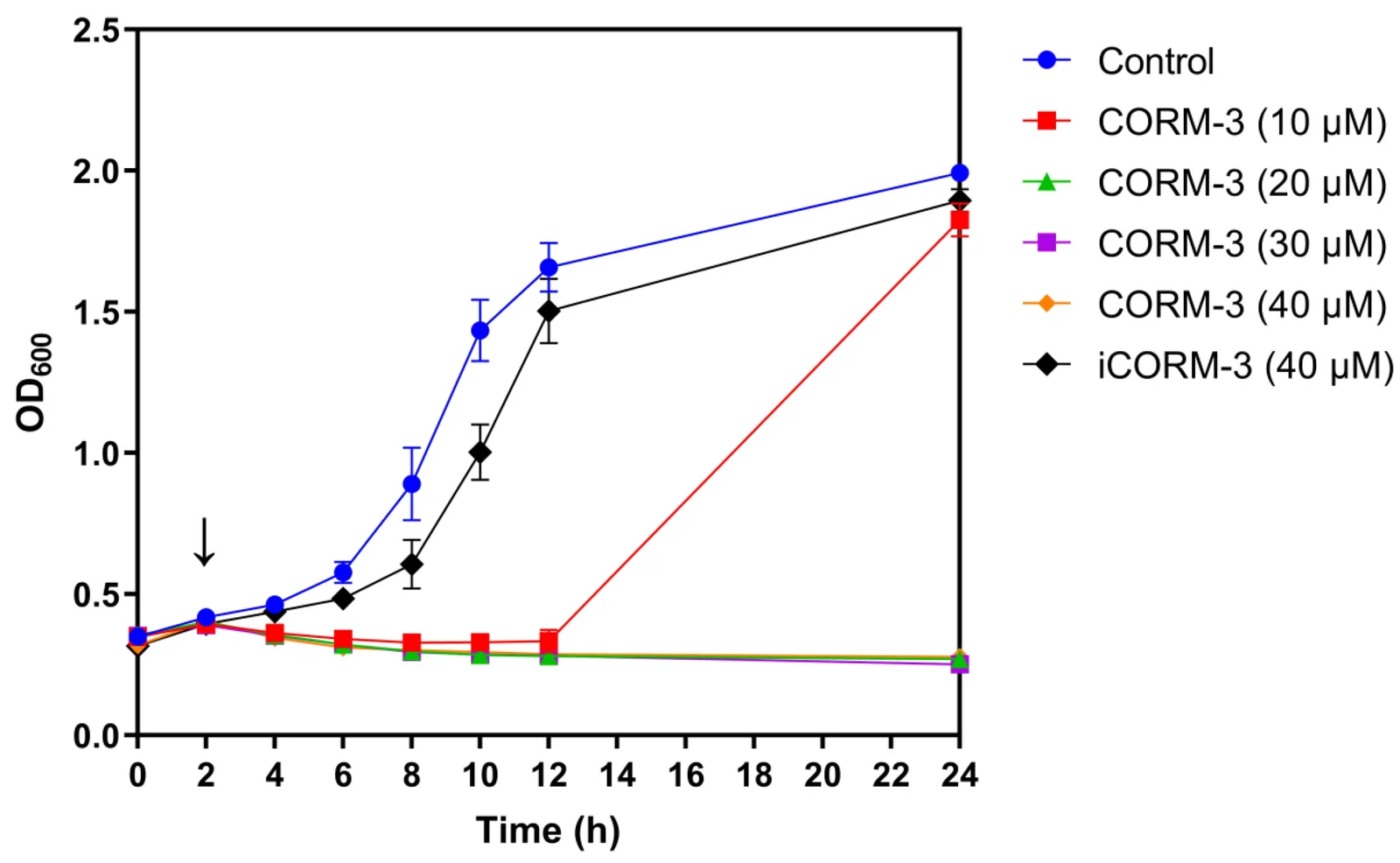
Growth kinetics of CORM-3 and iCORM-3 against *E. coli* MG1655. *E. coli* MG1655 was grown in a defined minimal medium in the presence of varying concentrations of CORM-3 and iCORM-3. The black arrow indicates the start of exposure. OD_600_ readings were taken at regular intervals over 24 h. Results are shown as mean ± SD from three independent biological replicates, each measured in duplicate (*n* = 6).

To investigate the effects of CORM-3 and iCORM-3 on *E. coli* MG1655 growth, we conducted a kinetic assay, and the results are shown in Figure 2.

CORM-3 inhibited bacterial growth for 12 h, regardless of the concentration tested. After this time, cells resumed growth only at 10 μM, reaching levels comparable to those of control, unexposed cultures, after 24 h of incubation. Conversely, complete growth inhibition was observed at 20 μM and 30 μM.

iCORM-3 did not exhibit antibacterial activity even at a higher concentration (40 μM), with a doubling time comparable to that of the unexposed control (11.1 vs. 12.5 h, respectively). Conversely, CORM-3 at 40 μM completely inhibited growth.

Based on these findings, 10 μM CORM-3 was considered a sub-inhibitory concentration for the proteomic experiment.

### 3.2. Global Proteome Profiling and Dataset Quality Control

Since CORM-3 showed antibacterial activity, we investigated its effects at the sub-inhibitory concentration of 10 μM in *E. coli* MG1655. Protein extracts were first assessed by one-dimensional SDS-PAGE before LC-MS/MS analysis (Supplementary Figure S1).

**Figure 3:**
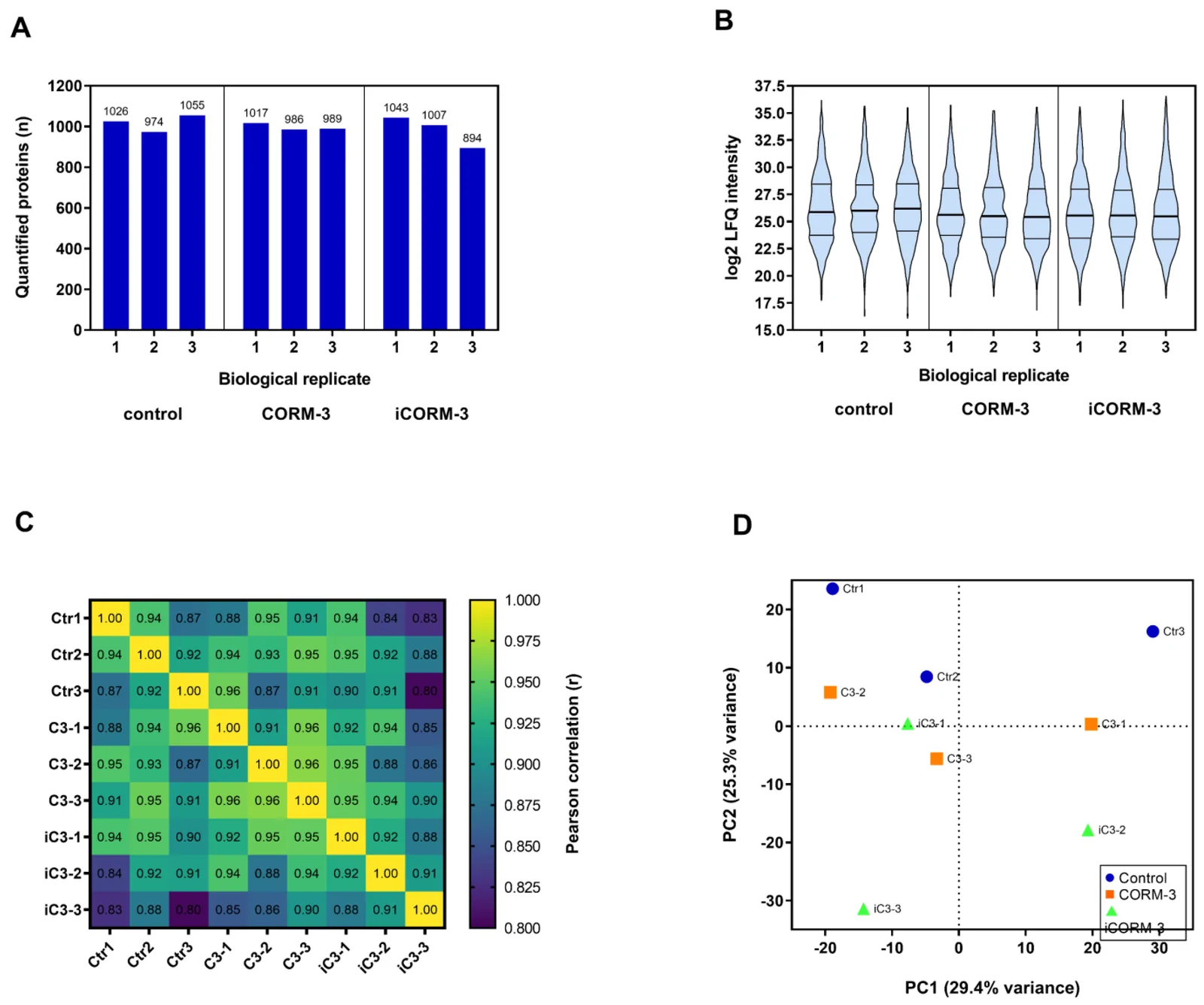
Quality control overview of the label-free quantitative proteomic dataset. (**A**) Number of quantified proteins detected in each biological replicate of untreated control, CORM-3-treated, and iCORM-3-treated *E. coli* MG1655 samples. (**B**) Violin and boxplot representation of log2-transformed LFQ intensity distributions across the nine samples. Median (bold line) and quartile (light lines) values are shown. (**C**) Pearson correlation heatmap of log2 LFQ intensities across all samples. Correlation coefficients are reported within each cell. (**D**) PCA of the proteomic profiles based on proteins quantified in at least five out of nine samples. Missing values were imputed using the median, and data were standardized before PCA. PC1 and PC2 explained 29.4% and 25.3% of the total variance, respectively. Control, CORM-3-treated, and iCORM-3-treated samples are indicated by different symbols.

A preliminary quality-control assessment was then performed to evaluate the robustness and comparability of the label-free proteomic dataset prior to differential abundance analysis (Figure 3).

Overall, 1322 proteins were included in the LFQ intensity matrix, providing broad proteome coverage suitable for exploratory comparative analysis. The number of quantified proteins was broadly comparable across the nine biological replicates, ranging from 974 to 1055 in control samples, from 986 to 1017 in CORM-3-treated samples, and from 894 to 1043 in iCORM-3-treated samples, with the lowest protein recovery observed in iCORM-3 replicate 3 (Figure 3A). Despite this lower recovery, the overall number of quantified proteins remained within an acceptable range and did not indicate a generalized loss of proteome coverage in the iCORM-3 group.

The distribution of log2-transformed LFQ intensities was largely comparable across all samples (Figure 3B). Pearson correlation analysis further supported the dataset’s overall comparability, showing generally high similarity among proteomic profiles, with correlation coefficients ranging from 0.80 to 0.96 (Figure 3C). These results indicate that the observed differences among samples were not primarily driven by major technical variation.

PCA performed on proteins quantified in at least five of nine samples showed that PC1 and PC2 explained 29.4% and 25.3% of the total variance, respectively. Although the experimental groups did not form completely distinct clusters, PCA revealed treatment-associated variability and suggested partial separation among control, CORM-3-treated, and iCORM-3-treated samples (Figure 3D). This pattern is consistent with the use of a sub-inhibitory CORM-3 concentration and suggests that treatment-associated effects were present but did not dominate the entire proteomic variance.

These QC results supported subsequent differential abundance and functional analyses.

### 3.3. Differential Abundance Analysis Reveals Bidirectional Proteomic Remodeling

**Figure 4:**
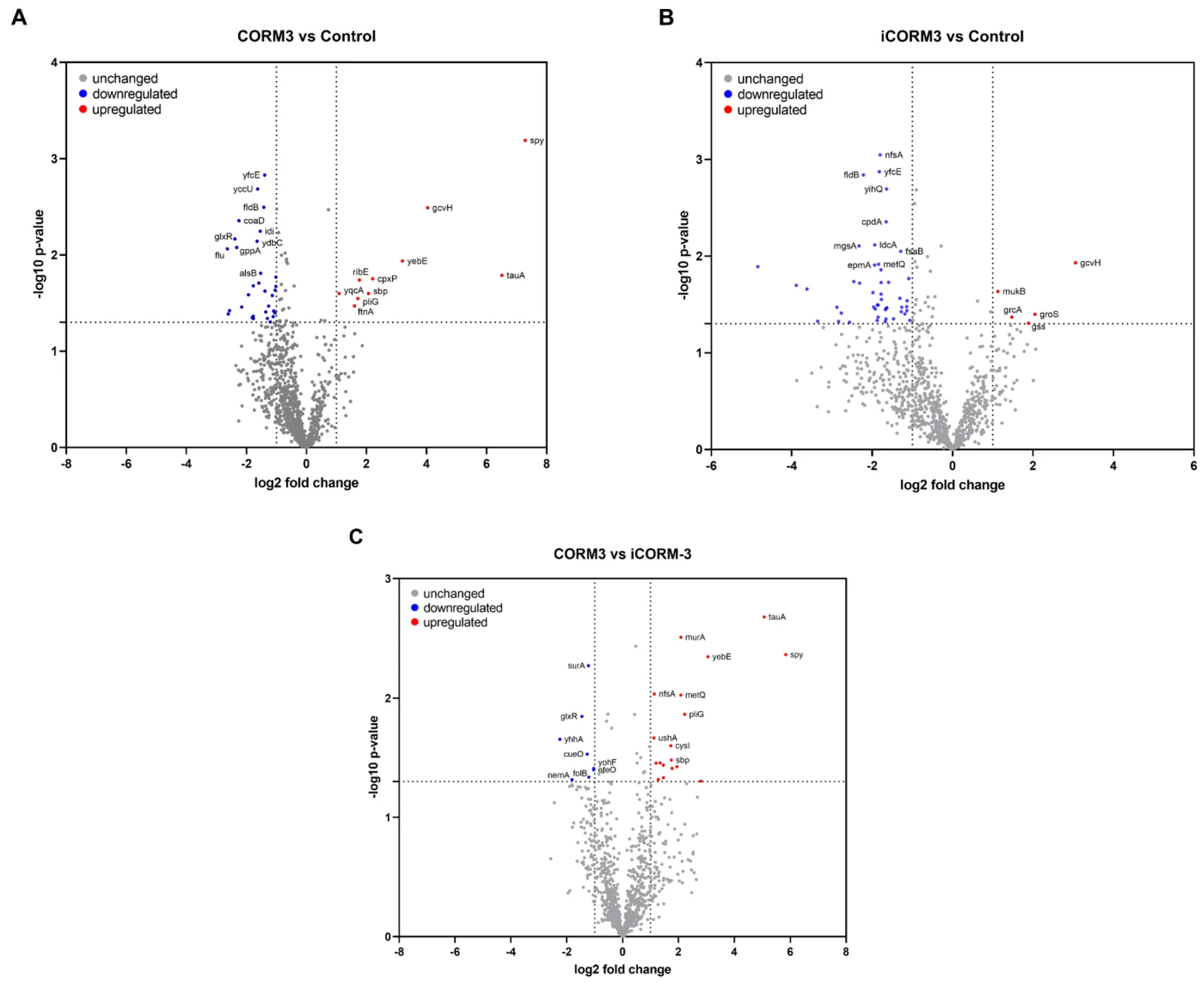
Differential abundance analysis of proteins detected in untreated, CORM-3-treated, and iCORM-3-treated *E. coli* MG1655 cells. Volcano plots showing pairwise protein abundance comparisons for (**A**) CORM-3 versus untreated control, (**B**) iCORM-3 versus untreated control, and (**C**) CORM-3 versus iCORM-3. For each protein, the x-axis reports the log2 fold change, and the y-axis reports the −log10 nominal *p*-value derived from Welch’s *t*-test. Vertical dashed lines indicate the two-fold change threshold, corresponding to log2FC = ±1, and the horizontal dashed line indicates *p* = 0.05. Proteins with nominal *p* < 0.05 and fold change ≥ 2 are shown as candidate increased proteins, whereas proteins with nominal *p* < 0.05 and fold change ≤ 0.5 are shown as candidate decreased proteins. *p*-values were adjusted for multiple testing using the Benjamini–Hochberg procedure; no protein reached FDR < 0.05. For better reading, only the top 10 proteins with the greatest treatment-associated changes are labeled.

Differential abundance analysis was performed to identify protein abundance changes associated with exposure to CORM-3 and to distinguish them from those induced by its inactive counterpart, iCORM-3 (Figure 4). Proteins meeting the exploratory threshold of nominal *p*-value < 0.05 and |log2FC| ≥ 1 were considered candidate differentially abundant proteins (candidate DAPs) and used for subsequent biological interpretation.

Three pairwise comparisons were analyzed: CORM-3 versus untreated control, iCORM-3 versus untreated control, and CORM-3 versus iCORM-3. In the CORM-3 versus control comparison, 41 candidate DAPs were identified, including 10 proteins with increased abundance and 31 with decreased abundance (Figure 4A). iCORM-3 treatment resulted in 52 candidate DAPs compared with untreated controls, including 5 increased and 47 decreased proteins (Figure 4B). The direct CORM-3 versus iCORM-3 comparison identified 26 candidate DAPs, with 18 proteins showing higher abundance and 8 showing lower abundance in CORM-3-treated cells (Figure 4C).

These results indicate that both compounds induced bidirectional proteomic changes, but with distinct abundance patterns. In particular, the direct comparison with iCORM-3 revealed a predominance of proteins with higher abundance in CORM-3-treated cells, suggesting that the active CO-releasing compound induced a proteomic signature only partially reproduced by the inactive comparator. Candidate proteins more abundant in CORM-3-treated cells included proteins related to envelope/periplasmic stress, sulfur metabolism, transport, and cell envelope-associated processes, such as Spy, TauA, YebE, CpxP, PliG, and MurA. Conversely, proteins with reduced abundance included candidates associated with energy metabolism, redox-related processes, metal homeostasis, and selected envelope or transport functions, such as SdhA, LpdA, GlxR, CueO, EfeO, and SurA.

To provide a focused overview of the most biologically informative changes, representative candidate DAPs from the main functional modules were summarized in Table 1. These included proteins associated with envelope/periplasmic stress, sulfur metabolism and transport, cell wall biogenesis, redox/metal homeostasis, and energy metabolism. Because no individual protein reached statistical significance after Benjamini–Hochberg correction for multiple testing (FDR < 0.05), these candidates should be interpreted as exploratory markers of CORM-3-associated proteomic remodeling rather than as definitive statistically significant protein changes.

**Figure 5:**
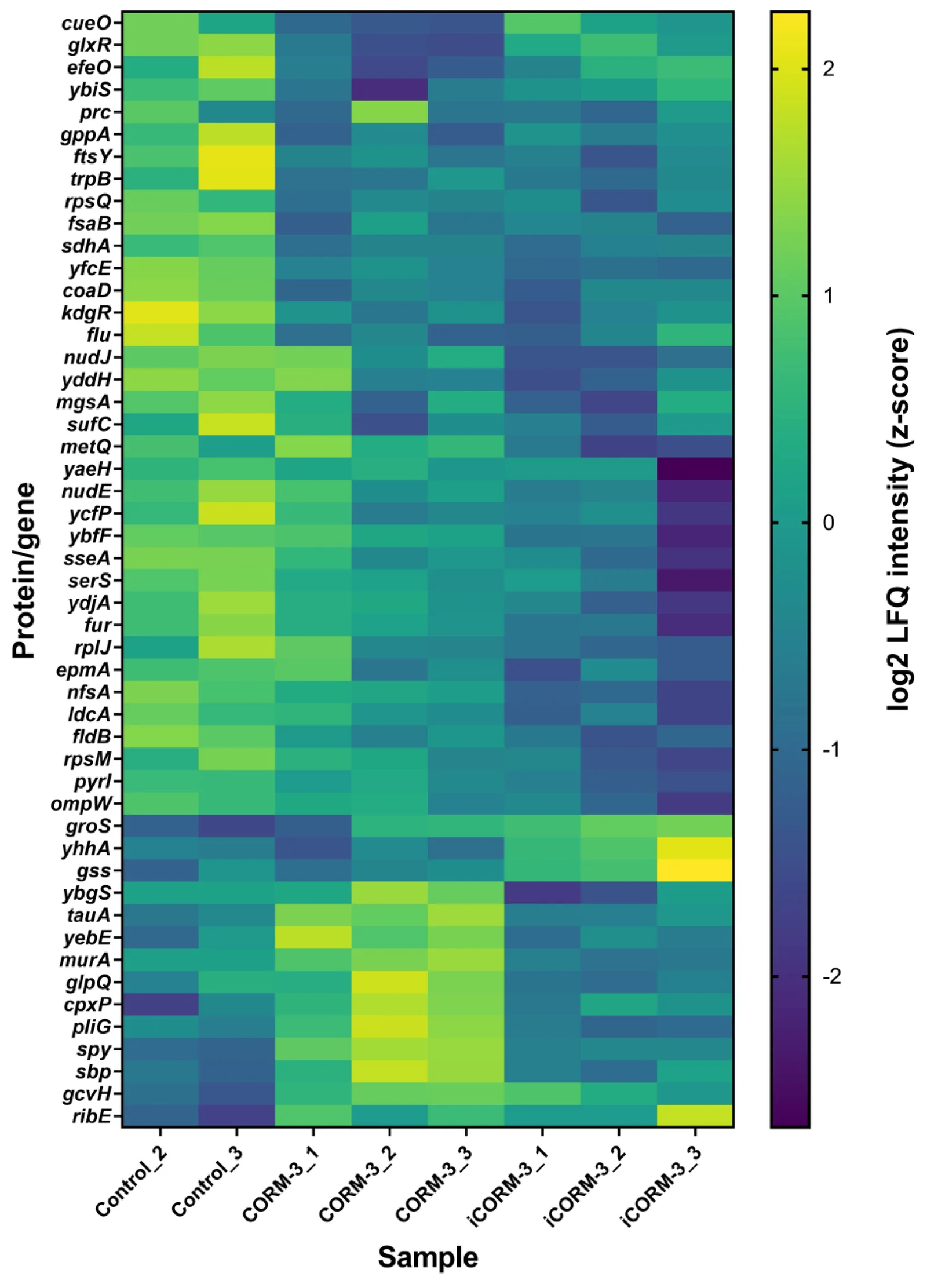
Heatmap of treatment-associated candidate differentially abundant proteins in *E. coli* MG1655. The heatmap displays the top 50 candidate DAPs selected based on a nominal *p*-value < 0.05 and |log2FC| ≥ 1 in at least one of the three pairwise comparisons: CORM-3 versus untreated control, iCORM-3 vs. untreated control, and CORM-3 vs. iCORM-3. LFQ intensities were log2-transformed and row-normalized as z-scores to emphasize relative within-protein abundance patterns across samples. Missing values were imputed using the row median for visualization purposes only. Rows represent proteins ordered by hierarchical clustering, whereas columns represent biological repli-cates grouped by experimental condition. Pairwise log2FC, nominal *p*-values and FDR values are reported in Table 1.

Heatmap, functional profiling, and network analysis identify treatment-associated modules. To visualize coordinated abundance patterns across experimental groups, a heatmap was generated using the top 50 candidate DAPs identified across the three pairwise comparisons (Figure 5). LFQ intensities were log2-transformed and row-normalized as z-scores. Proteins were selected based on a nominal *p*-value < 0.05 and |log2FC| ≥ 1 in at least one comparison, and ranked by the number and magnitude of treatment-associated changes.

The heatmap revealed distinct abundance patterns across untreated control, CORM-3-treated and iCORM-3-treated samples, with partial clustering according to treatment condition. CORM-3-treated cells showed a coordinated bidirectional profile, characterized by increased relative abundance of proteins involved in envelope/periplasmic stress, sulfur-related metabolism, transport, and adaptive responses, including Spy, TauA, YebE, CpxP, MurA, GlpQ, Sbp, and GcvH. Conversely, proteins with reduced relative abundance included candidates associated with central energy metabolism, redox/metal homeostasis, transport, and envelope remodeling. iCORM-3-treated cells displayed a partially overlapping but overall distinct profile, indicating that the inactive ruthenium-containing counterpart did not fully reproduce the proteomic signature induced by the active CO-releasing compound.

**Figure 6:**
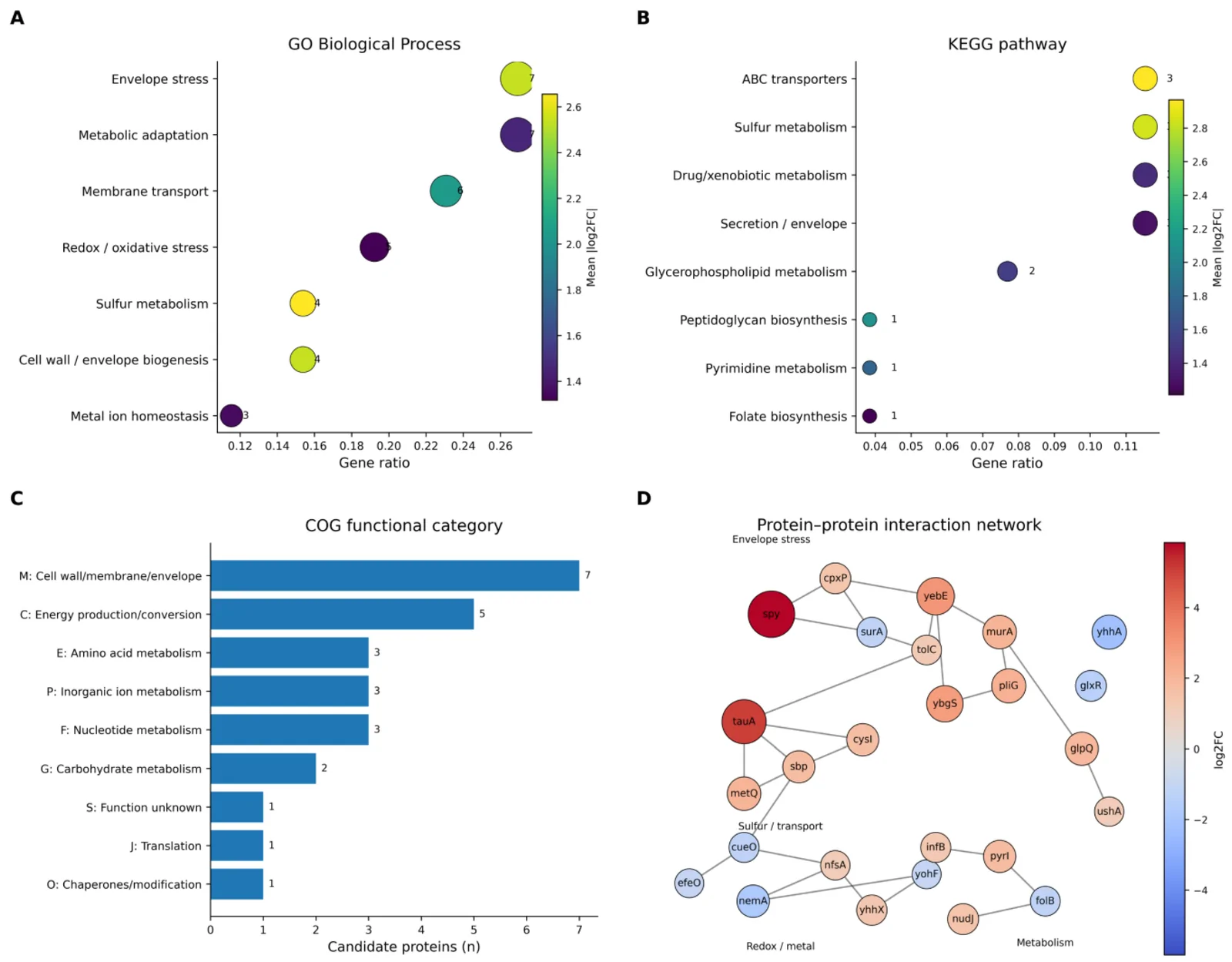
Functional profiling of CORM-3-associated candidate DAPs in *E. coli* MG1655. Candidate DAPs from the CORM-3 versus iCORM-3 comparison were analyzed using GO Biological Process grouping, KEGG pathway mapping, COG functional classification, and STRING-based protein–protein interaction analysis. Candidate DAPs were selected using a nominal *p*-value < 0.05 and |log2FC| ≥ 1. (**A**) GO Biological Process dot plot showing the main functional categories represented among candidate DAPs. (**B**) KEGG pathway dot plot showing the main associated pathways. (**C**) COG functional classification showing the distribution of candidate DAPs across broad functional categories. (**D**) STRING-based network of candidate DAPs from the CORM-3 vs. iCORM-3 comparison. Node color indicates log2FC, and node size reflects the magnitude of the change in abundance.

To determine whether these abundance patterns converged on specific biological functions, candidate DAPs from the CORM-3 versus iCORM-3 comparison were further analyzed using GO Biological Process grouping, KEGG pathway mapping, COG functional classification, and STRING-based protein–protein interaction analysis (Figure 6). GO grouping highlighted envelope stress, membrane transport, adaptive metabolic processes, sulfur metabolism, redox/oxidative stress, cell wall/envelope biogenesis, and metal ion homeostasis (Figure 6A). KEGG mapping identified pathways related to ABC transporters, sulfur metabolism, drug/xenobiotic metabolism, envelope-associated functions, glycerophospholipid metabolism, peptidoglycan biosynthesis, pyrimidine metabolism, and folate biosynthesis (Figure 6B). Consistently, COG classification indicated that candidate DAPs were mainly associated with cell wall/membrane/envelope biogenesis, energy production and conversion, amino acid transport and metabolism, inorganic ion transport, and nucleotide metabolism (Figure 6C). STRING-based visualization of the CORM-3 versus iCORM-3 candidate DAPs further supported the presence of functionally connected modules involving envelope/periplasmic stress, sulfur-related transport, redox/metal homeostasis, and metabolic adaptation (Figure 6D).

**Figure 7:**
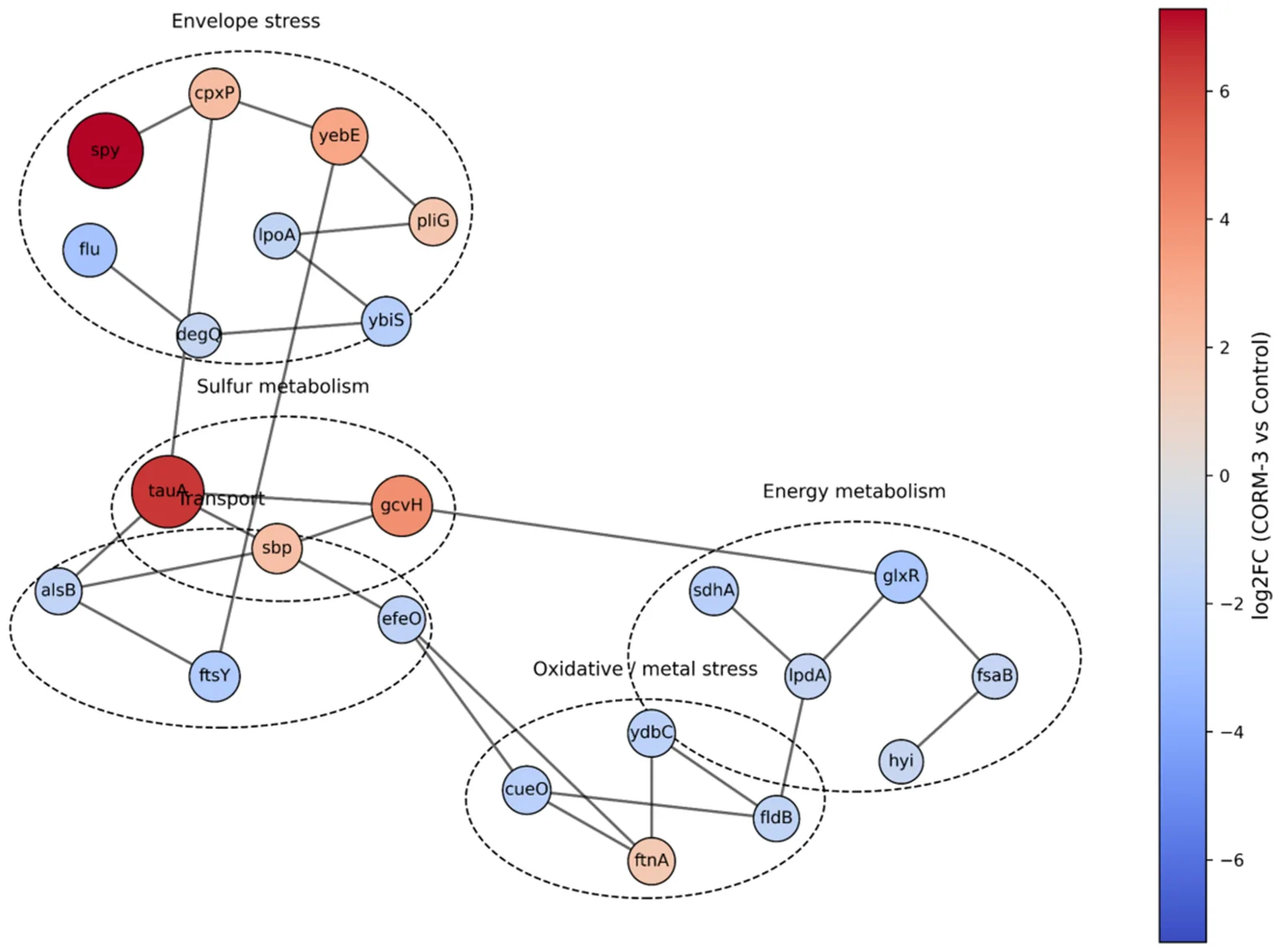
Network-based organization of CORM-3-responsive proteins in *E. coli* MG1655. STRING/Cytoscape-style network visualization of candidate DAPs identified in CORM-3-treated cells compared with untreated controls. Proteins were selected using a nominal *p*-value < 0.05 and |log2FC| ≥ 1. Nodes represent proteins, whereas edges indicate functional associations among proteins or within functional modules. Node color indicates the direction of abundance change, and node size is proportional to the absolute log2 fold change. Candidate DAPs were organized into five major functional modules: envelope stress, sulfur metabolism, transport, energy metabolism, and oxidative/metal stress. This network complements the CORM-3 vs. iCORM-3 analysis shown in Figure 6D by providing a broader view of the proteomic response induced by CORM-3 relative to untreated cells.

To complement this analysis, a second STRING/Cytoscape-style network was generated from candidate DAPs identified in the CORM-3 versus untreated control comparison (Figure 7). This broader network organized candidate DAPs into five modules—envelope stress, sulfur metabolism, transport, energy metabolism, and oxidative/metal stress—highlighting coordinated changes across stress-response and metabolic pathways.

Overall, heatmap, functional profiling, and network analyses indicate that CORM-3 exposure is associated with coordinated treatment-related modules rather than isolated protein abundance changes. These modules include both increased and decreased candidate DAPs, supporting a bidirectional proteomic remodeling involving envelope stress, sulfur-associated adaptation, redox/metal homeostasis, and central metabolic reprogramming.

### 3.4. Subcellular Localization of CORM-3-Responsive Proteins

To assess whether CORM-3-responsive proteins were preferentially associated with specific cellular compartments, candidate proteins identified in the CORM-3 vs. untreated control comparison were classified according to their annotated or predicted subcellular localization. Proteins were grouped into five categories: cytoplasm, inner membrane, outer membrane, periplasm, and extracellular/unknown categories.

**Figure 8:**
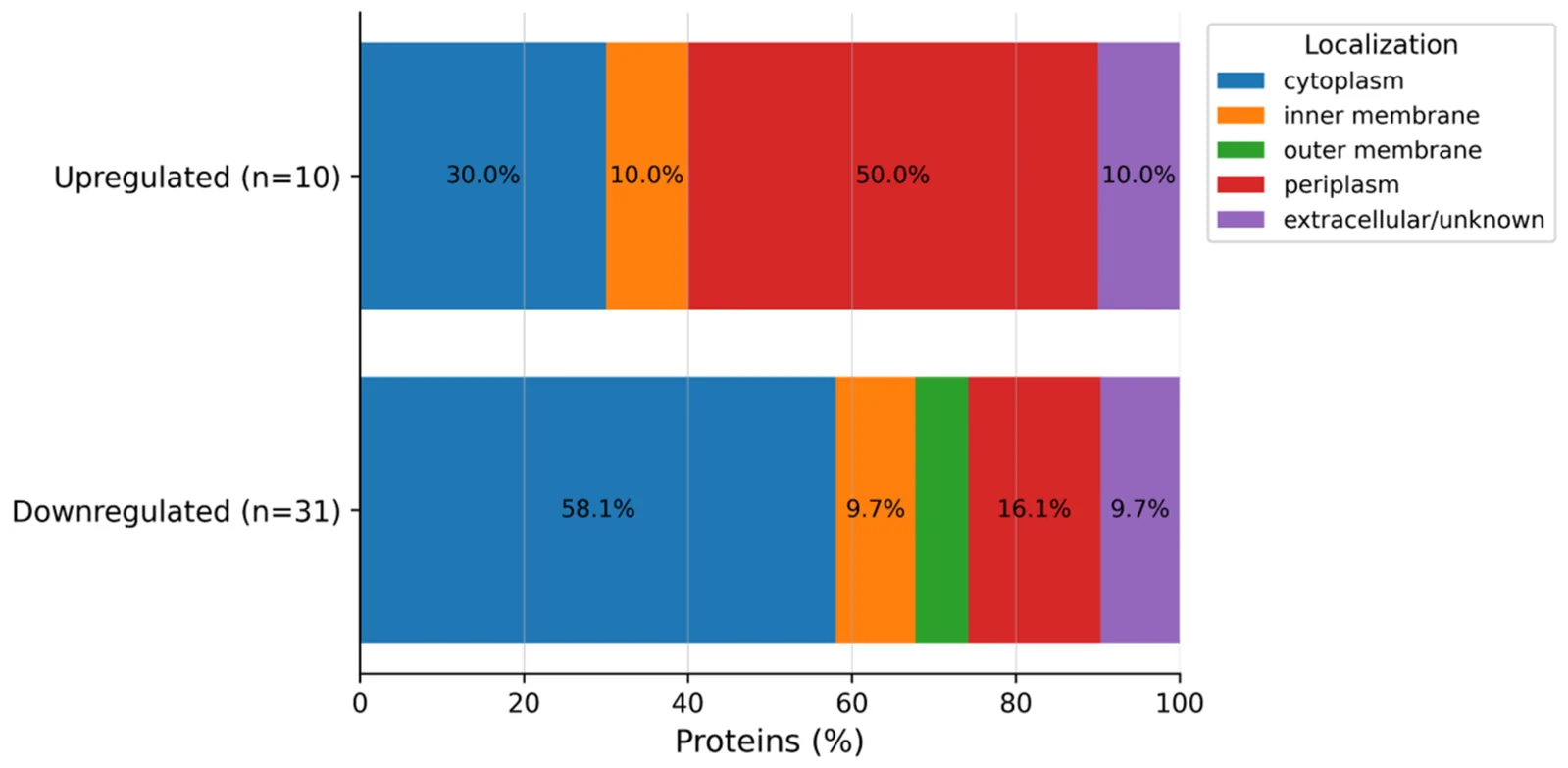
Subcellular localization of CORM-3-responsive proteins in *E. coli* MG1655. Candidate DAPs identified in CORM-3-treated cells compared with untreated controls were classified according to predicted or annotated subcellular localization. Proteins were grouped into five categories: cytoplasm, inner membrane, outer membrane, periplasm, and extracellular/unknown. The stacked barplot shows the percentage distribution of proteins with increased or decreased abundance across cellular compartments. Among increased proteins (*n* = 10), the counts were: cytoplasm, 3; inner membrane, 1; outer membrane, 0; periplasm, 5; extracellular/unknown. 1. Among decreased proteins (*n* = 31), the counts were: cytoplasm, 18; inner membrane, 3; outer membrane, 2; periplasm, 5; extracellular/unknown, 3. Periplasmic proteins represented the largest fraction of increased proteins, whereas decreased proteins were predominantly cytoplasmic, supporting enrichment of envelope- and periplasm-associated responses among proteins increased by CORM-3 exposure. Data were from a total-proteome LFQ-based analysis and should therefore be interpreted as evidence of an envelope/periplasm-associated candidate signature.

This analysis revealed a clear difference in the compartmental distribution of proteins with increased and decreased abundance after CORM-3 exposure (Figure 8).

Among proteins with increased abundance (*n* = 10), the periplasm represented the most frequent localization, accounting for 50.0% of the total, followed by the cytoplasm (30.0%), inner membrane (10.0%), and extracellular/unknown category (10.0%); no outer membrane proteins were detected among the increased candidates. In contrast, proteins with decreased abundance (*n* = 31) were predominantly cytoplasmic (58.1%), with smaller fractions assigned to the periplasm (16.1%), inner membrane (9.7%), outer membrane (6.5%), and extracellular/unknown category (9.7%).

These findings indicate that CORM-3 exposure is associated with compartment-specific, bidirectional proteomic remodeling. The relative enrichment of periplasmic proteins among increased candidates is consistent with activation of envelope-associated stress responses and periplasmic adaptation. In contrast, the predominance of cytoplasmic proteins among decreased candidates supports broader metabolic attenuation or reprogramming. Thus, subcellular localization analysis reinforces the view that CORM-3 affects both cell envelope-associated stress pathways and intracellular metabolic functions. Because this analysis was based on total-proteome LFQ data rather than membrane-enriched fractions, it should be interpreted as evidence of an envelope/periplasm-associated candidate signature rather than a comprehensive assessment of the membrane proteome.

Taken together, differential abundance, heatmap, functional annotation, network, and subcellular localization analyses suggest that sub-inhibitory CORM-3 exposure is associated with a candidate, bidirectional proteomic signature in *E. coli* MG1655. Although no individual protein reached FDR < 0.05, candidate DAPs converged on envelope/periplasmic stress, sulfur-related transport and metabolism, redox/metal homeostasis, and central energy metabolism, with a less evident signature in iCORM-3-treated cells.

## 4. Discussion

The functional characterization of bacterial responses to CORMs provides an important step toward understanding their potential as antimicrobial agents and clarifying their mechanisms of action. Building on previous work and our recent findings on the specific interactions of these molecules with commonly used antibiotics [17], the present study provides a proteomic analysis of *E. coli* MG1655 exposed to a sub-inhibitory concentration of Ru(CO)_3_Cl (glycinate) (CORM-3). This approach allowed us to explore how bacterial cells remodel protein-level pathways in response to CORM-3 and to distinguish, at least in part, responses associated with the active CO-releasing compound from those elicited by the CO-depleted control.

Growth kinetics experiments confirmed that CORM-3 exerted a clear concentration-dependent inhibitory effect, whereas its inactive counterpart, iCORM-3, did not exhibit this activity, even at higher concentrations. This observation confirms previous studies indicating that CORM-3 and related CORMs display antibacterial activity against Gram-negative bacteria, including *E. coli* [5,10,11,12,18]. At the same time, the marked difference between CORM-3 and iCORM-3 supports the biological relevance of the active CO-releasing compound and provides a useful experimental framework for interpreting downstream proteomic changes. However, this distinction should not be interpreted as evidence that the antibacterial activity of CORM-3 is exclusively mediated by CO release. Indeed, CORM-3 biology remains complex, as thiol-reactive ruthenium species have been reported to contribute substantially to its antimicrobial and cytotoxic properties [13]. Therefore, the direct comparison between CORM-3 and iCORM-3 is a critical aspect of the present study, as it helps identify responses more closely associated with the active compound while accounting for potential effects of the residual Ru-containing scaffold.

Notably, the proteomic signatures identified in this study provide valuable insights into the ongoing discussion regarding the primary cellular drivers of CORM-3 activity. Recent studies, most notably by Southam et al. [13], have proposed that the toxicity of CORM-3 toward bacteria may depend substantially on a thiol-reactive ruthenium species rather than exclusively on intracellular carbon monoxide delivery. Our findings in defined minimal medium under sub-inhibitory exposure (10 µM) are remarkably consistent with this ruthenium/thiol-centered framework. The robust modulation of sulfur acquisition and transport components (TauA, Sbp, MetQ), cysteine/sulfite processing machinery (CysI/J), metal homeostasis factors (FtnA, CueO, EfeO), and periplasmic chaperones (Spy, CpxP) strongly suggests that the Ru-containing scaffold exerts direct reactivity toward cellular thiols, periplasmic protein targets, and metal-binding networks. Interestingly, despite candidate shifts in nucleotide metabolism, major canonical DNA repair pathways (e.g., LexA/RecA-mediated SOS response) were not significantly induced in our dataset. This observation suggests that at mild sub-inhibitory concentrations, envelope stress buffering, metal sequestration, and thiol/sulfur metabolic adaptation represent the primary front-line defenses prior to the onset of systemic genomic damage. Future functional studies incorporating extracellular cysteine or thiol-rescue assays will be pivotal to further dissect the precise interplay between Ru-mediated electrophilic toxicity and CO-dependent physiological signaling.

Consistently, comparative proteomic analysis revealed that CORM-3 exposure was associated with a broader and more functionally coherent response than iCORM-3. Candidate proteins modulated after CORM-3 treatment converged on several interconnected processes, including envelope and periplasmic stress, sulfur metabolism and transport, redox and metal homeostasis, and central energy metabolism. Although these changes did not reach statistical significance after multiple-testing correction, their functional convergence supports the interpretation that CORM-3 elicits a coordinated bacterial stress response rather than affecting a single isolated cellular target.

Importantly, the CORM-3-associated proteomic signature was not limited to increased protein abundance. In the CORM-3 versus untreated control comparison, candidate-decreased proteins outnumbered candidate-increased proteins, indicating that the response to CORM-3 also involves attenuation or remodeling of selected cellular functions. This aspect is biologically relevant because reduced protein abundance may reflect decreased synthesis, increased turnover, or a shift in cellular priorities during stress adaptation. In particular, proteins with reduced abundance were enriched among cytoplasmic and metabolic functions, including SdhA, LpdA, GlxR, FsaB, and Hyi, suggesting that CORM-3 exposure is associated with remodeling of central carbon metabolism, redox-linked enzymatic reactions, and respiratory energy production. This pattern is consistent with the growth lag observed after CORM-3 exposure and supports the hypothesis that *E. coli* may transiently reduce energy-intensive metabolic activities while activating protective envelope, sulfur, and redox-associated responses.

One of the most coherent signatures emerging from the proteomic analysis was the involvement of periplasmic and cell envelope-associated proteins. The bacterial envelope is a major stress-sensing compartment, and envelope stress responses, including the Cpx system, are central to detecting and managing misfolded envelope proteins, envelope integrity perturbations, and defects in protein trafficking [19,20,21]. In the present study, CORM-3 exposure was associated with increased abundance of several envelope- and periplasm-associated candidates, including Spy, CpxP, YebE, PliG, and TauA, as well as modulation of proteins involved in envelope biogenesis and maintenance, such as SurA and TolC. This pattern is consistent with perturbation of envelope-associated functions and suggests activation of periplasmic protein quality-control pathways among the subset of proteins detected by the present LFQ workflow.

Among these candidates, Spy represented one of the most prominent CORM-3-associated proteins. Spy is a periplasmic chaperone-like protein involved in the stabilization of unfolded or misfolded proteins under envelope stress conditions. Its marked increase in CORM-3-treated cells, relative to both untreated controls and iCORM-3-treated cells, strongly supports activation of periplasmic protein quality-control mechanisms. This response is biologically consistent with the parallel increase in CpxP, a periplasmic component and modulator of the Cpx envelope stress pathway. CpxP can act as a sensor and negative regulator of CpxA under basal conditions. At the same time, its induction is generally associated with envelope stress and accumulation of misfolded proteins in the periplasm [20]. Therefore, the concomitant modulation of Spy and CpxP suggests that CORM-3 exposure induces a periplasmic stress condition that requires chaperone-mediated stabilization and Cpx-dependent adaptation.

Additional candidate proteins reinforce this interpretation. YebE, an inner membrane-associated protein, may reflect perturbation of membrane-associated functions, whereas PliG, a periplasmic inhibitor of g-type lysozyme, points to activation of envelope-protective mechanisms. SurA modulation is also relevant, as it is a major periplasmic chaperone involved in the folding and assembly of outer membrane proteins [22]. Changes in SurA, together with modulation of TolC, an outer membrane efflux channel involved in envelope transport and multidrug efflux, suggest that CORM-3 exposure affects not only periplasmic protein quality control but also outer membrane maintenance and transport-associated envelope functions. Overall, these findings support the view that envelope remodeling and periplasmic stress adaptation represent early and central components of the bacterial response to CORM-3.

At the same time, the envelope-associated response also included proteins with reduced abundance, indicating that CORM-3 does not simply induce a generalized upregulation of envelope proteins. Reduced abundance of proteins such as SurA, together with decreases in selected envelope-associated candidates identified in the network analysis, including LpoA, YbiS, DegQ, and Flu, suggests a broader remodeling of envelope biogenesis, protein folding, and surface-associated functions. This pattern may reflect selective reorganization of the envelope proteome, in which stress-response and protective proteins increase, while other envelope-maintenance or surface-associated proteins decrease. Therefore, the envelope signature should be interpreted as adaptive remodeling rather than uniform activation of all envelope-related functions.

Beyond the cell envelope, proteomic analysis suggested that CORM-3 also affects metabolic pathways related to sulfur acquisition, sulfur-containing amino acid metabolism, and redox homeostasis. Sulfur-containing molecules and thiol-dependent systems are central to bacterial redox buffering, protein repair, and protection against oxidative and electrophilic stress, and their modulation is consistent with a broader adaptive response to CORM-3-induced damage [23]. In this context, the increased abundance of TauA is particularly informative. TauA is the periplasmic binding component of the taurine transport system and is involved in the uptake of taurine and related sulfonates as sulfur sources, particularly under sulfur-limiting conditions [24]. Its marked increase suggests that CORM-3 exposure may create a cellular state in which sulfur acquisition becomes functionally relevant.

The modulation of Sbp and MetQ further supports this interpretation. Sbp is a periplasmic sulfate-binding protein involved in sulfate transport, whereas MetQ is a methionine-binding lipoprotein implicated in methionine uptake [25]. Together, TauA, Sbp, and MetQ indicate that CORM-3 exposure affects multiple entry points for sulfur-containing nutrient acquisition, including sulfonate-, sulfate-, and methionine-related pathways. Downstream of uptake, the modulation of CysI and CysJ, components of sulfite reductase, suggests involvement of sulfur assimilation and cysteine biosynthesis. Although these changes do not demonstrate depletion of intracellular sulfur-containing metabolites, they support the hypothesis that CORM-3 increases the cellular demand for sulfur-containing compounds required for thiol-dependent redox buffering, protein repair, and maintenance of iron-sulfur cluster-containing enzymes.

The increased abundance of GcvH adds a further metabolic dimension. GcvH is the lipoyl-bearing H protein of the glycine cleavage system, which catalyzes glycine degradation and transfers one-carbon units to tetrahydrofolate, thereby linking glycine catabolism to folate-dependent biosynthetic pathways [26]. Because one-carbon metabolism is closely linked to methionine biosynthesis, modulation of GcvH may reflect broader metabolic rewiring connecting amino acid metabolism, sulfur homeostasis, and redox balance. Overall, the coordinated involvement of TauA, Sbp, MetQ, CysI/CysJ, and GcvH supports the idea that sulfur and amino acid homeostasis are central components of *E. coli*’s adaptive response to CORM-3.

These findings are consistent with the previous transcriptomic analysis reported by McLean et al. [14], which showed that CORM-3 and iCORM-3 elicit distinguishable bacterial responses and identified sulfur-containing species as major components of CORM-3 action. In the present proteomic dataset, this relationship was most evident at the functional-module level, with candidate changes involving TauA, Sbp, MetQ and CysI/CysJ pointing to perturbation of sulfonate, sulfate and methionine/cysteine-related pathways. The modulation of GcvH further links this response to amino acid, one-carbon and redox-associated metabolism. Direct transcript–protein concordance should be interpreted cautiously because the two studies differ in exposure conditions, timing, analytical platform and biological readout. Nevertheless, the convergence of sulfur-associated and stress-adaptation themes supports the biological plausibility of the candidate proteomic signature identified here.

The changes observed in proteins involved in energy metabolism, redox homeostasis, and metal homeostasis provide an additional mechanistic layer that links CORM-3 exposure to respiratory and oxidative stress. CO can interact with transition-metal centers and heme-containing proteins, making bacterial respiratory chains plausible targets of CO-dependent stress. In this context, the reduced abundance of SdhA is particularly relevant because succinate dehydrogenase links the tricarboxylic acid cycle to the respiratory electron transport chain. A decrease in SdhA may therefore reflect attenuation or remodeling of respiratory metabolism during the growth lag phase induced by CORM-3. Similarly, reduced abundance of LpdA, a dihydrolipoyl dehydrogenase involved in central carbon metabolism and redox reactions, may indicate altered flux through dehydrogenase-dependent pathways. The decrease in GlxR, associated with glyoxylate-related redox metabolism, together with the reduced abundance of FsaB and Hyi, further supports the interpretation that CORM-3 exposure affects carbon flux organization and redox-associated enzymatic functions. Rather than representing isolated losses of individual proteins, these decreases suggest a coordinated metabolic downshift or reallocation of cellular resources in response to CORM-3-induced stress.

Redox and metal-associated proteins provide complementary information. CueO, a periplasmic multicopper oxidase involved in copper/redox homeostasis, and EfeO, a component associated with iron uptake [25], both showed reduced abundance, suggesting altered management of periplasmic metal-dependent processes and iron acquisition under CORM-3 stress. In contrast, the increased abundance of FtnA, a bacterial non-heme ferritin involved in iron storage, suggests activation of iron-sequestration pathways that may help limit the availability of free iron for metal-catalyzed oxidative reactions. NfsA, an oxygen-insensitive NADPH-dependent nitroreductase, further suggests changes in redox and detoxification capacity. Taken together, these findings are consistent with previous evidence that CORM-3 and related CO-releasing molecules can affect bacterial respiration, global transcriptional regulators, and reactive oxygen species-mediated killing [12,18], as well as with the broader concept that oxidative stress in bacteria targets proteins, metal cofactors, and metabolic enzymes and requires coordinated repair and protective responses [23,27].

**Figure 9:**
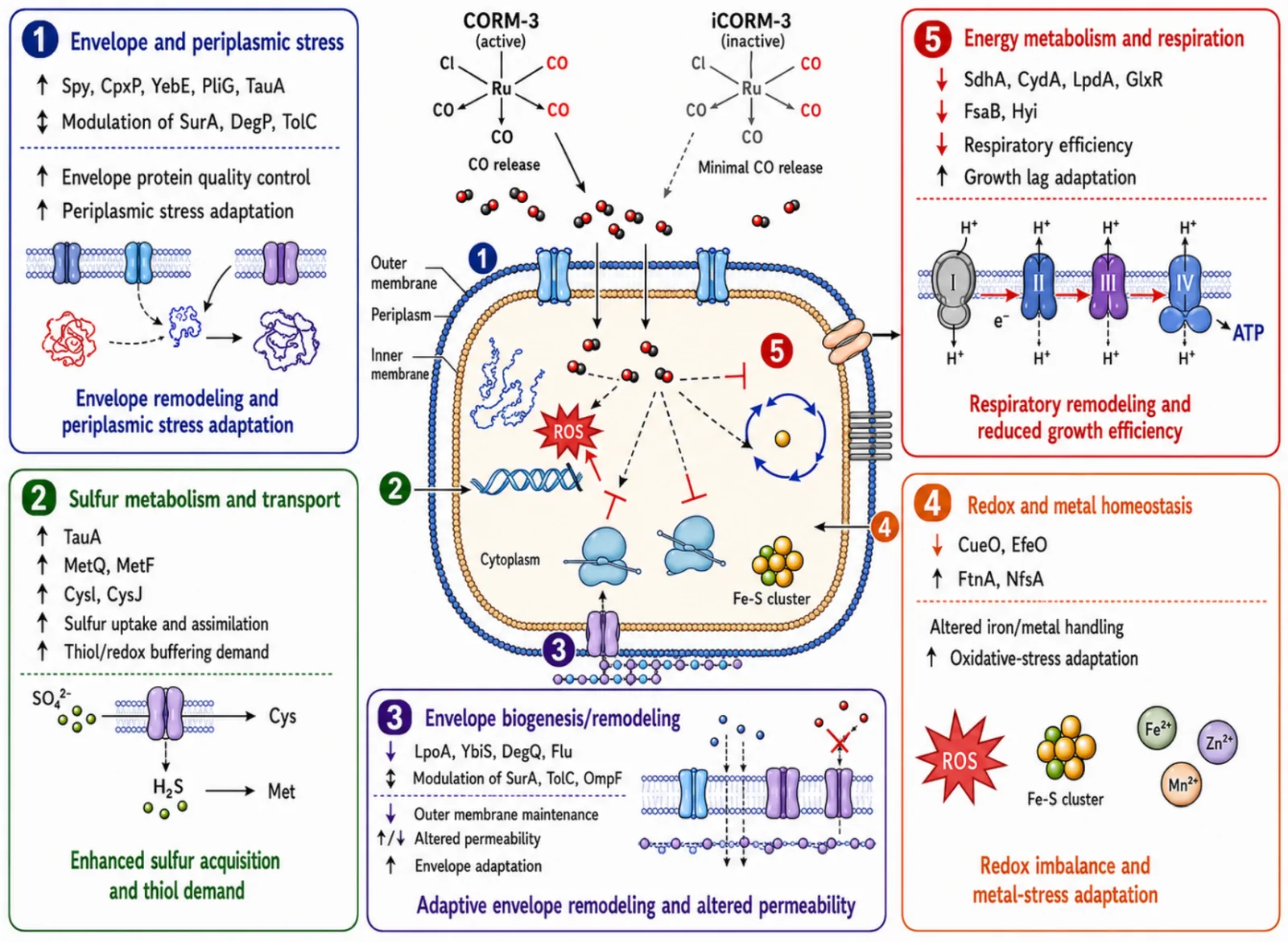
Exploratory model of the candidate proteomic response of *E. coli* MG1655 to CORM-3 exposure. The schematic summarizes candidate proteomic signatures associated with sub-inhibitory CORM-3 exposure compared with iCORM-3. Candidate abundance changes converged on envelope/periplasmic stress, sulfur metabolism and transport, redox/metal homeostasis, and energy metabolism/respiration. Overall, the model supports a hypothesis-generating framework in which CORM-3 exposure is associated with multifactorial bacterial stress adaptation involving activation of stress-protective pathways and attenuation of selected metabolic functions. Because no individual protein reached statistical significance after multiple-testing correction and no independent validation was performed, this model should be regarded as exploratory and requiring targeted validation. Colors identify the five functional modules represented in the model: blue, envelope and periplasmic stress; green, sulfur metabolism and transport; purple, envelope biogenesis/remodeling; orange, redox and metal homeostasis; and red, energy metabolism and respiration. Upward and downward arrows indicate increased and decreased protein abundance or functional activity, respectively. Solid arrows represent proposed processes or functional relationships, whereas dashed arrows indicate indirect or hypothesized effects. Red T-shaped lines denote inhibitory effects.

These interconnected processes are summarized in the proposed mechanistic model shown in Figure 9, which integrates the main candidate proteomic signatures associated with CORM-3 exposure in *E. coli* MG1655.

This study shows some limitations. First, no individual protein reached statistical significance after multiple-testing correction, likely reflecting the exploratory nature of the LFQ proteomic design, the limited number of biological replicates, and the moderate magnitude of the effects induced by sub-inhibitory CORM-3 exposure. Second, the experimental layout does not capture strain-dependent, medium-dependent, dose-dependent, or time-dependent proteomic remodeling. Third, using total protein extracts combined with SDS-PAGE/in-gel digestion may underrepresent highly hydrophobic, low-abundance, or tightly membrane-associated proteins; therefore, the envelope/periplasm-associated signature reported here should not be interpreted as a comprehensive membrane-proteome analysis. Fourth, although iCORM-3 represents an essential CO-depleted comparator, this experimental design cannot fully disentangle the relative contributions of CO release and Ru-dependent reactivity. In addition, cysteine- or thiol-based rescue experiments were not performed; such approaches will be important in future studies to assess whether extracellular thiol scavenging attenuates CORM-3 activity and the associated pro-teomic response, thereby helping to clarify the relative contributions of Ru-dependent reactivity and CO release to CORM-3 activity. Finally, independent validation of selected candidate proteins was not performed; future studies should verify key components of the proposed signature, such as Spy/CpxP, TauA/Sbp/MetQ, CueO/FtnA and SdhA/LpdA, using targeted proteomics, immunoblotting, transcriptional reporters or mutant-based approaches.

## 5. Conclusions

In conclusion, this exploratory LFQ proteomic study provides a protein-level perspective on the response of *E. coli* MG1655 to sub-inhibitory CORM-3 exposure. Compared with iCORM-3, CORM-3 was associated with candidate abundance changes converging on envelope/periplasmic stress, sulfur-acquisition and metabolism, redox/metal homeostasis, and selected energy-associated functions. Although no individual protein reached FDR significance, the convergence of candidate DAPs across interconnected cellular processes supports a hypothesis-generating model in which CORM-3 exposure is associated with multifactorial bacterial stress adaptation, rather than a single dominant protein-level target. These findings are consistent with previous transcriptomic evidence and suggest that sulfur-containing pathways, envelope homeostasis, respiratory/redox adaptation, and attenuation of selected energy-associated functions represent candidate components of the bacterial response to CORM-3. Future studies integrating targeted proteomics, metabolomics, thiol-redox profiling, DNA-damage assay, and functional validation of selected candidate proteins will be required to clarify the relative contribution of CO release and Ru-dependent reactivity to CORM-3 antibacterial activity.

## Figures and Tables

**Table 1:** Key CORM-3-responsive proteins selected for biological interpretation. Proteins were selected based on biological relevance (e.g., envelope/periplasmic stress, sulfur metabolism and transport, cell wall biogenesis, redox/metal homeostasis, and energy metabolism) and on representativeness of the major CORM-3-associated proteomic signatures. For each protein, the following are reported: gene name, protein annotation, main biological function, subcellular localization, log2 fold change values for CORM-3 vs. untreated control and CORM-3 vs. iCORM-3 comparisons, nominal *p*-values, and Benjamini–Hochberg FDR values. Because no protein reached an FDR < 0.05 after multiple-testing correction, these proteins should be interpreted as candidate treatment-responsive proteins to support functional and mechanistic interpretation.

Gene	Protein	Main Function	Localization	log2FC CORM-3/Control	*p*/FDR CORM-3/Control	log2FC CORM-3/iCORM-3	*p*/FDR CORM-3/iCORM-3
*spy*	Spheroplast protein Y	Periplasmic stress/chaperone-like envelope response	Periplasm	7.28	6.47 × 10^−4^/0.459	5.84	0.004/0.830
*tauA*	Taurine-binding periplasmic protein	Taurine/sulfonate transport; sulfur acquisition	Periplasm	6.51	0.016/0.672	5.07	0.002/0.830
*yebE*	Inner membrane protein YebE	Envelope-associated inner membrane protein	Inner membrane	3.20	0.012/0.580	3.05	0.005/0.830
*cpxP*	Periplasmic protein CpxP	Cpx envelope stress response modulator	Periplasm	2.21	0.018/0.672	1.46	0.036/0.869
*sbp*	Sulfate-binding protein	Sulfate transport; sulfur metabolism	Periplasm	2.07	0.025/0.676	1.75	0.033/0.869
*pliG*	Inhibitor of g-type lysozyme	Envelope protection/cell wall-associated defense	Periplasm	1.71	0.028/0.676	2.22	0.014/0.869
*metQ*	D-methionine-binding lipoprotein MetQ	Methionine uptake; amino acid/sulfur metabolism	Periplasm	0.25	0.560/0.872	2.09	0.009/0.869
*murA*	UDP-N-acetylglucosamine 1-carboxyvinyltransferase	Peptidoglycan biosynthesis/cell wall precursor synthesis	Cytoplasm	1.86	0.087/0.676	2.08	0.003/0.830
*gcvH*	Glycine cleavage system H protein	One-carbon metabolism; glycine cleavage system	Cytoplasm	4.03	0.003/0.459	0.98	0.195/0.869
*glpQ*	Glycerophosphoryl diester phosphodiesterase	Glycerophospholipid metabolism; phosphate release	Periplasm	1.61	0.066/0.676	1.94	0.038/0.869
*cysI*	Sulfite reductase [NADPH] hemoprotein beta-component	Sulfite reduction; cysteine/sulfur metabolism	Cytoplasm	1.07	0.180/0.724	1.72	0.025/0.869
*nfsA*	Oxygen-insensitive NADPH nitroreductase	Redox activity; nitroreduction/detoxification	Cytoplasm	−0.67	0.012/0.580	1.13	0.009/0.869
*tolC*	Outer membrane protein TolC	Outer membrane efflux channel; envelope transport	Outer membrane	0.55	0.117/0.676	1.20	0.035/0.869
*cueO*	Blue copper oxidase CueO	Copper/redox homeostasis; oxidative stress-related function	Periplasm	−1.77	0.021/0.672	−1.27	0.029/0.869
*efeO*	Iron uptake system component EfeO	Iron uptake/metal ion transport	Periplasm	−1.58	0.020/0.672	−1.04	0.040/0.869
*ftnA*	Bacterial non-heme ferritin	Iron storage/metal homeostasis	Cytoplasm	1.60	0.034/0.676	0.60	0.512/0.894
*sdhA*	Succinate dehydrogenase flavoprotein subunit	TCA cycle/respiratory energy metabolism	Inner membrane	−1.80	0.045/0.676	0.05	0.887/0.970
*lpdA*	Dihydrolipoyl dehydrogenase	Central carbon metabolism; redox enzyme	Cytoplasm	−1.30	0.046/0.676	0.50	0.513/0.894
*glxR*	2-hydroxy-3-oxopropionate reductase	Carbon metabolism; glyoxylate-related redox reaction	Cytoplasm	−2.38	0.007/0.569	−1.46	0.014/0.869
*surA*	Chaperone SurA	Periplasmic chaperone; outer membrane protein folding	Periplasm	−0.61	0.155/0.714	−1.22	0.005/0.830

## Data Availability

The original contributions presented in this study are included in the article/Supplementary Materials. Further inquiries can be directed to the corresponding authors.

## Supplementary Materials

The following supporting information can be downloaded at: https://www.mdpi.com/article/10.3390/microorganisms14091918/s1, Supplementary Figure S1: One-dimensional SDS-PAGE of protein extracts from *E. coli* MG1655 after CORM-3 or iCORM-3 treatment. Ladder: molecular weight standard. Lanes 1–3: untreated controls. Lanes 4–6: cells exposed to CORM-3 at 10 μM. Lanes 7–9: cells exposed to iCORM-3 at 10 μM.
