# Supplementary material for: Exploratory Label-Free Proteomic Profiling of the Escherichia coli Response to Sub-Inhibitory CORM-3 Exposure
Source: Microorganisms. 2026 Aug 31;14(9):1918. doi: 10.3390/microorganisms14091918 (PMC13608947; doi:10.3390/microorganisms14091918)
Supplement: Supplementary file 1 [file microorganisms-14-01918-s001.zip › microorganisms-4510835-supplementary.pdf]

SUPPLEMENTARY MATERIALS

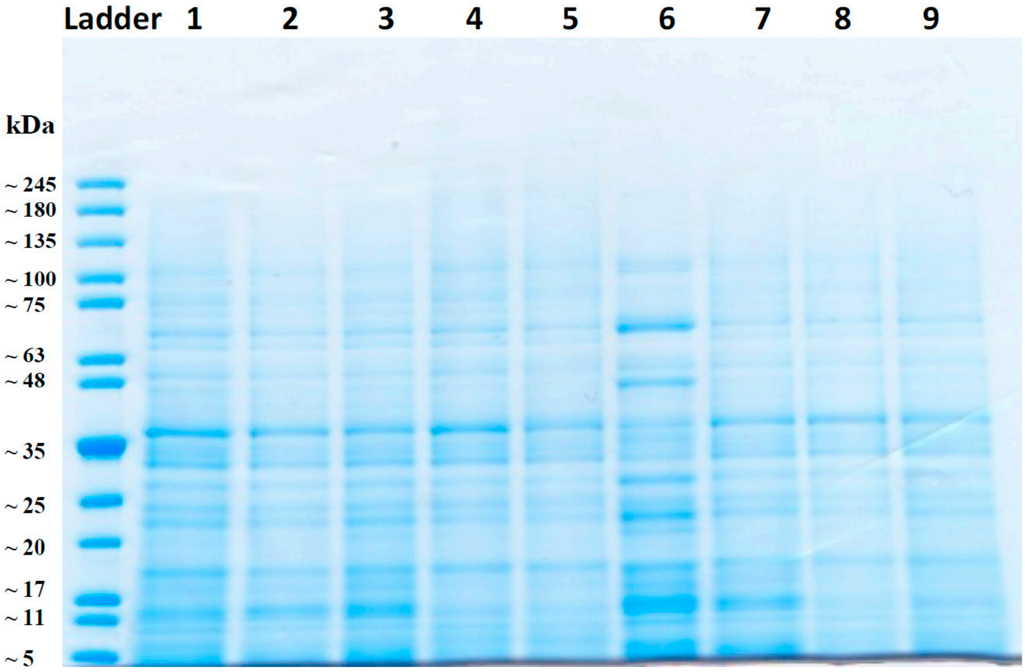

Supplementary Figure S1. One-dimensional SDS-PAGE of protein extracts from *E. coli* MG1655 after CORM-3 or iCORM-3 treatment. Ladder: molecular weight standard. Lanes 1–3: untreated controls. Lanes 4–6: cells exposed to CORM-3 at 10 µM. Lanes 7–9: cells exposed to iCORM-3 at 10 µM.
